# Supplementary material for: The invasive New Guinea flatworm Platydemus manokwari in France, the first record for Europe: time for action is now
Source: PeerJ. 2014 Mar 4;2:e297. doi: 10.7717/peerj.297 (PMC3961122; doi:10.7717/peerj.297)

Traduction intégrale en Français de l'article :

The invasive New Guinea flatworm *Platydemus manokwari* in France, the first record for Europe: time for action is now

Jean-Lou Justine, Leigh Winsor, Delphine Gey, Pierre Gros, Jessica Thévenot

Publié dans : **PeerJ, 2014**

DOI 10.7717/peerj.297

Le ver plat de Nouvelle-Guinée *Platydemus manokwari* en France, première mention en Europe : il faut agir maintenant

Jean-Lou Justine, Leigh Winsor, Delphine Gey, Pierre Gros, Jessica Thévenot

Jean-Lou Justine, ISYEB, Institut de Systématique, Évolution, Biodiversité, UMR7205 CNRS, EPHE, MNHN, UPMC, Muséum National d'Histoire Naturelle, CP 51, 55 rue Buffon, 75231 Paris cedex 05, France

Leigh Winsor, School of Marine and Tropical Biology, James Cook University, Townsville Qld 4811, Australia

Delphine Gey, UMS 2700 Service de Systématique moléculaire, Muséum National d'Histoire Naturelle, CP 26, 57 rue Cuvier, 75231 Paris cedex 05, France

Pierre Gros, Naturaliste Amateur, 26 Route de France, 06800 Cagnes-sur-Mer, France

Jessica Thévenot, Coordination technique et scientifique de la stratégie nationale relative aux espèces exotiques envahissantes, Service du Patrimoine Naturel, Muséum National d'Histoire Naturelle, CP 41, 36 rue Geoffroy Saint-Hilaire, 75231 Paris cedex 05, France

Auteur pour la correspondance : Jean-Lou Justine, justine@mnhn.fr

Résumé

Des vers plats (Plathelminthes) terrestres non-indigènes ont été signalés dans treize pays européens. Ils comprennent *Bipalium kewense* et *Dolichoplana striata*, qui sont en grande partie limités aux serres et que l'on peut considérer comme des espèces non-invasives. Les autres espèces viennent de l'hémisphère sud, comme le ver plat de Nouvelle-Zélande *Arthurdendyus triangulatus*, invasif au Royaume-Uni, en Irlande et aux îles Féroé, le ver plat australien *Australoplana sanguinea alba* en Irlande et au Royaume-Uni, et le ver plat australien bleu des jardins *Caenoplana coerulea* en France, à Minorque et au Royaume-Uni. Le Royaume-Uni a une douzaine ou plus d'espèces non-indigènes dont la plupart sont des espèces australiennes et néo-zélandaises. Ces espèces peuvent passer à un stade invasif lorsque des conditions environnementales optimales et autres sont rencontrées, et les vers plats ont alors la possibilité de causer un préjudice économique ou environnemental. Dans cet article, nous rapportons l'identification, à partir de la morphologie et de l'analyse moléculaire des séquences COI, de vers plats terrestres non-indigènes trouvés dans une serre à Caen (France) comme étant le ver plat de Nouvelle-Guinée *Platydemus manokwari* de Beauchamp, 1963 (Plathelminthes, Continenticola, Geoplanidae, Rhynchodeminae). *Platydemus manokwari* fait partie des « 100 espèces exotiques envahissantes les plus néfastes au monde ». Des listes des mentions géographiques dans le monde, des proies sur le terrain et des proies en laboratoire sont fournies pour *P. manokwari*. Cette espèce est considérée comme une menace pour les escargots indigènes partout où elle est introduite. La découverte récente de *P. manokwari* en France représente une extension significative de la distribution de cette espèce exotique envahissante de la région Indo-Pacifique à l'Europe. Au cas où s'échapperait de la serre, le ver plat pourrait survivre aux hivers et s'établir dans les pays tempérés. L'existence de cette espèce en France nécessite une alerte précoce de cette incursion aux autorités des États et de l'Union européenne, suivie par l'éradication de l'espèce dans sa localité, le resserrement des mesures internes de quarantaine pour prévenir la propagation de l'espèce à partir de et vers ce site, l'identification, si possible, de l'origine probable du ver plat, et le traçage d'autres incursions éventuelles pouvant résulter de la dispersion accidentelle de plantes et du sol du site.

Introduction

Une conséquence indésirable de la mondialisation, un phénomène relativement moderne, a été une augmentation du nombre des invasions biologiques qui menacent la conservation de la biodiversité et des ressources naturelles (Secrétariat de NOBANIS 2012 ; Simberloff 2014). Les espèces exotiques envahissantes (EEE) ont été définies comme « les plantes, les animaux, les pathogènes et autres organismes qui ne sont pas indigènes à un écosystème, et qui peuvent causer un préjudice économique ou environnemental, ou nuire à la santé humaine. En particulier, elles ont un impact défavorable sur la diversité biologique, y compris le déclin ou l'élimination des espèces indigènes (grâce à la compétition, la prédation ou la transmission d'agents pathogènes), et sur la perturbation des écosystèmes locaux et des fonctions des écosystèmes » (Convention sur la diversité biologique 2009).

Les invasions biologiques historiques comprennent la dispersion passive de vers plats terrestres, aussi appelés « planaires terrestres » ou Plathelminthes terrestres. Le principal moteur de ceci a été probablement les horticulteurs du 19ème siècle qui utilisaient les boîtes de Ward, alors récemment inventées, pour transporter en toute sécurité vers les serres et les jardins de l'Europe des plantes rares, avec un sol contenant des espèces animales exotiques cryptiques (Winsor et al. 2004). En conséquence plus de 30 espèces de Plathelminthes terrestres se sont imposées comme espèces non-indigènes dans divers pays en dehors de leur aire de répartition naturelle (Winsor et al. 2004).

Dans les habitats modifiés par l'homme, les Plathelminthes et leurs cocons continuent à être associés à des plantes à racines et rhizomes, en pot, et certains types de légumes frais (Alford et al. 1996). Une dispersion secondaire ultérieure de ces espèces de Plathelminthes exotiques se produit à travers l'échange et l'achat de plantes de pépinières, les jardins botaniques, les centres de jardinage et les jardiniers (Alford et al. 1996), en particulier les pépinières infestées et les centres de jardinage (Boag et al 1994 ; Moore et al. 1998), la dispersion accidentelle active à travers les traditions sociales d'échanges de plantes et le recyclage de la couche arable (Christensen & Mather, 1998), ou par l'introduction délibérée de vers plats pour des fins de contrôle biologique d'une espèce de nuisible tels que l'escargot géant africain *Achatina fulica* Bowdich 1822 dans la région Pacifique (Muniappan 1987 ; Waterhouse & Norris 1987).

Les Plathelminthes terrestres sont des carnivores qui se nourrissent d'une variété d'organismes du sol tels que les vers de terre, les isopodes, les insectes et les escargots. Certains Plathelminthes EEE peuvent constituer une menace pour la biodiversité locale (Alford et al. 1996 ; Cowie 2010 ; Santoro et Jones, 2001 ; Ducey et al. 2007 ; Sugiura 2010) et avoir un impact négatif sur l'agriculture, par exemple par un déclin des espèces des vers de terre (Murchie et Gordon 2013) résultant en une baisse de fertilité des sols (Murchie 2010) et, éventuellement, du drainage (Jones et al, 2001).

Des Plathelminthes terrestres non-indigènes ont été signalés dans treize pays européens (Filella-Subirá 1983 ; Jones, 1998 ; Kawakatsu et al., 2002 ; Ogren et al. 1997). Ces vers plats peuvent être divisés en deux grands groupes : les « anciennes » et les « nouvelles » espèces introduites.

Le groupe des « anciennes espèces » comprend *Bipalium kewense* Moseley, 1878 et *Dolichoplana striata* Moseley 1877 qui ont été sans aucun doute introduites par inadvertance en Europe au 19ème siècle par les horticulteurs. L'une ou l'autre de ces espèces sont présentes dans dix pays européens, et sont les seuls vers plats terrestres non-autochtones actuellement connues en Autriche, Belgique, République tchèque, Finlande, Allemagne, Norvège, Pologne et Portugal. Ces deux espèces semblent être largement limitées à des serres en Europe et ne répondent pas aux critères ci-dessus des espèces exotiques envahissantes : les espèces sont très répandues, mais ne forment des populations localisées. Toutefois, ces espèces peuvent passer à un stade invasif lorsque des conditions optimales environnementales et autres sont présentes, et les vers plats ont alors le potentiel d'avoir un impact sur la faune du sol, en particulier les vers de terre comme cela s'est produit dans des régions de l'Amérique du Nord avec *Dolichoplana striata* (Hyman 1954) et des espèces de *Bipalium* (Ducey et al 2005, 2007).

Le groupe des « nouvelles espèces » de vers plats non-indigènes présentes en Europe comprend principalement des espèces de l'hémisphère sud comme le ver plat de Nouvelle-Zélande *Arthurdendyus triangulatus* (Royaume-Uni, Irlande, îles Féroé), le ver plat australien *Australoplana sanguinea alba* (Moseley, 1877) (Irlande, Royaume-Uni) et le ver plat australien bleu des jardins *Caenoplana coerulea* Moseley, 1877 (Royaume-Uni, France, et plus récemment Minorque (Breugelmans et al. 2012) et Espagne (Mateos et al. 2013)). Le Royaume-Uni a une douzaine ou plus d'espèces non-indigènes dont la plupart sont des espèces australiennes et néo-zélandaises (Jones, 2005).

Nous avons récemment identifié des vers plats terrestres non-indigènes trouvés dans une serre à Caen (France) comme étant le ver plat de Nouvelle Guinée *Platydemus manokwari* de Beauchamp, 1963. L'identité de ces vers plats a ensuite été confirmée par analyse moléculaire des séquences de COI. *Platydemus manokwari* fait partie des «100 pires espèces exotiques envahissantes du monde » (Lowe et al. 2000). Dans cet article, nous présentons des preuves de l'identification de l'espèce en France, qui est la première mention en Europe, une brève liste des mentions de l'espèce dans le monde, des listes de ses proies connues, et les options possibles pour son contrôle.

Matériel et Méthodes

Matériel

Les spécimens ont été trouvés dans une serre du Jardin des Plantes à Caen (France) ; selon des témoins, il est probable que des spécimens similaires étaient présents dans la serre depuis des mois. Les spécimens ont été prélevés à la main et envoyés vivant à Paris par service postal. Huit spécimens ont été traités. Cinq ont été gardés en vie et utilisés pour des expériences de prédation et des photographies détaillées ; ils sont morts après plusieurs jours et ont été détruits. Trois spécimens ont été tués dans de l'eau chaude, puis stockés dans de l'éthanol (spécimens JL81A et JL81B) ou du formol (JL81C). Un petit morceau du corps a été prélevé chez les deux individus fixés à l'éthanol pour l'analyse moléculaire. Les photographies ont été transmises à l'un d'entre nous (LW) pour identification. Une description anatomique histologique n'a pas été entreprise. Les spécimens sont déposés dans les collections du Muséum National d'Histoire Naturelle, Paris, sous le numéro d'enregistrement MNHN JL81A-C.

Des expériences de prédations limitées ont été menées avec les quelques spécimens vivants disponibles. Très simplement, les vers plats ont été mis dans un petit récipient en plastique avec des escargots vivants.

Séquences moléculaires

L'ADN génomique a été extrait à partir d'un petit morceau de ver, à l'aide du kit QIAamp DNA Mini (Qiagen). Un fragment de 424 paires de bases du gène COI a été amplifié avec les amorces COI- ASmit1 (avant 5'- TTTTTTGGGCATCCTGAGGTTTAT – 3') et CI - ASmit2

(inverse 5'- TAAAGAAAGAACATAATGAAAATG - 3') (Littlewood et al., 1997). La réaction PCR a été réalisée dans 20 µl, contenant 1 ng d'ADN, du tampon 1X CoralLoad PCR, MgCl2 3 mM, 66 µM de chaque dNTP, 0.15µM de chaque amorce et 0,5 unités de Taq DNA polymérase (Qiagen). Le protocole d'amplification était de : 4' à 94 °C, suivi de 40 cycles de 94 °C pendant 30'', 48 °C pendant 40'', 72 °C pendant 50'', avec une extension finale à 72 °C pendant 7'. Les produits de PCR ont été purifiés et séquencés dans les deux sens sur un séquenceur DNA Analyzer 3730xl à 96 capillaires (Applied Biosystems). Les séquences ont été vérifiées avec le logiciel CodonCode Aligner (CodonCode Corporation, Dedham, MA, USA), comparées à base de données GenBank en utilisant BLAST et déposées dans GenBank sous le numéro d'enregistrement KF887958. Les séquences ont été comparées en utilisant MEGA5 (Tamura et al. 2011).

Résultats

Morphologie

Le ver plat est plus large au milieu, et s'amincit régulièrement vers l'avant mais plus brusquement en arrière (Figure 1). Deux grands yeux proéminents sont situés en retrait de la pointe de la tête, qui est en forme de bec allongé (Figure 2). En coupe transversale le ver plat est convexe dorsalement et plat ventralement. Le spécimen vivant et viable figuré ici est long de 50 mm de et large de 5 mm. La bouche est située juste en arrière du milieu du ventre, avec le gonopore à mi-chemin entre la bouche et l'extrémité postérieure. Le dos est d'une couleur brun olive foncé, et sous grossissement montre un grain brun pâle. Une bande dorsale longitudinale médiane, crème pâle, approximativement large de 0,3 mm, commence juste derrière les yeux et continue jusqu'à l'extrémité postérieure (Figures 1 et 3). La couleur brun olive passe au gris à l'extrémité antérieure. Une mince bande submarginale crème avec une marge grisâtre fine court latéralement le long de la longueur du corps à partir de l'extrémité antérieure (Figure 3). La surface ventrale est un brun clair finement tacheté de plus clair (Figures 4-5), légèrement plus pâle à mi-longueur. Ces caractéristiques sont compatibles avec celles de *Platydemus manokwari* de Beauchamp, 1963 (Plathelminthes, Continenticola, Geoplanidae, Rhynchodeminae) (de Beauchamp, 1962 ; Kawakatsu et al. 1992 ; Winsor 1990).

Expériences de prédation

Divers escargots vivants ont été introduits dans le même récipient qu'un ver plat. Quatre des cinq spécimens sont morts avant de se nourrir. Un événement de prédation unique a été observé, sur l'Helicidae *Eobania vermiculata* Müller. Le pharynx cylindrique, dépassant de la surface ventrale, était visible lorsque le ver plat s'attaquait à l'escargot (Figure 5).

Identification moléculaire

Les deux séquences COI que nous avons obtenues à partir de deux individus étaient identiques. Elles ont été comparées aux seules séquences de COI disponibles d'un membre du genre *Platydemus* dans GenBank (*Platydemus manokwari*, numéro d'accession : AF178320.1). La p-distance entre notre nouvelle séquence et la séquence GenBank de *Platydemus manokwari* était de 4%. Bien que ce ne soit pas mentionné dans GenBank, nous savons que ce spécimen a été récolté en Australie par l'un de nous (LW). La population à partir de laquelle le spécimen de GenBank de *Platydemus manokwari* (AF178320.1) a été prélevé avait déjà été confirmé histologiquement (par LW, à partir du lot LW1065) comme appartenant à l'espèce *P. manokwari* et des spécimens ont été déposés dans le musée du Queensland (numéros d'enregistrement GL4724, spécimen entiers dans l'alcool, et GL4725, 126 lames).

Clarification nomenclaturale

Il existe une certaine variation dans la littérature sur la date de description de *P. manokwari*, 1962 ou 1963. Nous avons examiné attentivement la publication originale. Le document a été présenté lors d'une réunion en décembre 1962 et est inclus dans le volume de 1962, mais la date de publication effective est le 18 Avril 1963. En accord avec l'article 21.1 (Commission Internationale de Nomenclature Zoologique 1999), la date du taxon est 1963. La date bibliographique de la publication reste 1962, mais le taxon est : *Platydemus manokwari* de Beauchamp, 1963.

Discussion

Identification moléculaire

La p-distance entre nos deux séquences et la séquence GenBank de *Platydemus manokwari* est de 4%. Cette distance génétique correspond approximativement aux distances génétiques généralement observées entre espèces étroitement apparentées ou entre populations éloignées à l'intérieur d'une seule espèce. Alvarez-Presas et al. (2012) ont étudié la variation du COI chez des espèces appartenant à la même famille que *Platydemus manokwari*, c'est à dire les Geoplanidae. Dans cette étude de l'espèce européenne *Microplana terrestris* (Müller, 1774), les échantillons ont été étudiés à partir de deux localités, à l'Est et à l'Ouest du Nord de l'Espagne. Les variations allaient de 0 % à 3 % dans les localités de l'Ouest, et de 0% à 1,6% dans les localités de l'Est ; l'Ouest et l'Est présentaient une différence de 2,4 % à 4 %. La différence entre espèces (*M. terrestris* vs *M. robusta* Vila- Farré et Sluys, 2011) était d'environ 19 %. Par conséquent, nous considérons que la différence de 4 % constatée entre nos spécimens français de *P. manokwari* et l'échantillon australien dans GenBank est compatible avec une variation intraspécifique. Les données moléculaires confirment donc l'identification morphologique.

Mentions précédentes de Plathelminthes terrestres en France

Les mentions précédentes de Plathelminthes terrestres non-indigènes en France comprennent un *Pelmatoplana* sp. dans une serre à Saint Max, banlieue de Nancy (identifiés par de Beauchamp *in* Remy 1942), *Bipalium kewense* et *Caenoplana coerulea* dans un jardin urbain à Villeneuve-de-la-Raho, département des Pyrénées-Orientales (mentionnés comme "France" dans Winsor et al. 2004), où des plantes en pot achetées dans un supermarché locale de plantes ont été considérées comme l'origine des vers plats (Gérard Peaucellier, *in litt.*), et *Bipalium kewense* à Orthez et Bayonne, département des Pyrénées-Atlantiques (Vivant 2005).

D'autres espèces, souvent non identifiées, ont été signalées récemment en France dans des journaux (Gasiglia 2013 ; Guyon 2014 ; Heyligen 2013), des magazines (Groult & boucourt 2014) et un blog (Justine 2013), et mentionnées dans des documents gouvernementaux (Placé 2013), mais pas dans des réelles publications scientifiques.

L'apparition du Plathelminthe invasif *Platydemus manokwari* dans le Jardin des Plantes, à Caen, dans le département de Basse-Normandie (Normandie, France), est la première mention de l'espèce en Europe.

Mentions précédentes de *Platydemus manokwari*

Platydemus manokwari est présent à la station Pindaunde, Mont Wilhelm, à 3625 mètres d'altitude (de Beauchamp, 1972 ; Winsor 1990), où il a été trouvé sous des pierres avec *Platydemus longibulbus* de Beauchamp, 1972 et *Platydemus pindaude*i de Beauchamp, 1972, et à Kainantu, à 1558 mètres d'altitude, dans les montagnes de l'Est de la Nouvelle-Guinée (Winsor 1990). L'aire de répartition naturelle de cette espèce de montagne n'a pas encore été déterminée.

Jusqu'à présent, *Platydemus manokwari* était confinée à la région Indo-Pacifique dans les limites, au nord, des îles Ogasawara (Japon), au sud, de Mackay dans le Queensland (Australie), à l'est, de la Polynésie Française, et avec l'extension la plus occidentale du ver dans les Maldives. La découverte à Caen de cette espèce est une extension significative vers l'ouest de la présence de *P. manokwari*, à partir de la Région Indo-Pacifique jusqu'à l'Europe.

Depuis qu'il a été découvert dans la Station de recherche agricole à Manokwari, Irian Jaya en 1962 où on lui a attribué le déclin de l'escargot géant africain *Achatina fulica*, un ravageur envahissant des plantations de noix de coco et d'autres cultures (Mead, 1963 ; Mead, 1979 ; Muniappan et. al 1986 ; Schreurs 1963 ; van Driest 1968 ; Waterhouse & Norris 1987), *Platydemus manokwari* s'est progressivement répandu dans l'Indo-Pacifique (Tableau 1, Figure 6). Le ver plat a été accidentellement introduit, probablement avec des plantes et des sols, dans différentes îles de la région Pacifique, notamment l'Australie, Guam, Palau, Hawaii, les États fédérés de Micronésie, la Polynésie Française et Samoa. La mention la plus récente de *P. manokwari* dans la région du Pacifique est sa présence dans l'archipel des Fidji à Rotuma (Brodie et al. 2013). L'espèce a également été délibérément introduite comme agent de lutte biologique contre l'escargot géant africain *Achatina fulica* à Bugsuk aux Philippines (Muniappan et al. 1986 ; Waterhouse & Norris 1987), Yokohama, Japon (Eldredge & Smith, 1995), et aux Maldives (Muniappan 1987). Le taux de dispersion secondaire de *Platydemus manokwari* est faible et dépend du transport par les humains de plants infectés et de sol, ou des vers plats eux-mêmes. L'espèce semble être incapable de parcourir de longues distances toute seul : il lui a fallu 12 mois pour coloniser des habitats de jardins urbains mixtes séparés par quelques 30 mètres de pelouse (Winsor, 1990), et à Fua Mulaku (Maldives), et il lui a fallu un an pour éliminer *Achatina* dans un rayon de 180 mètres autour du site de relâchage (Muniappan 1987).

Reproduction

Dans des conditions expérimentales, la température optimale pour l'élevage de *P. manokwari*, en termes de longueur de pré-oviposition et de production de cocons, est 24 °C, avec une période de développement post-ponte moyen (entre ponte et sortie du cocon) pour les jeunes de 7,8 ± 1,2 jours (Kaneda et al. 1992). Les cocons contiennent une moyenne de 5,2 (3–9) juvéniles chacun. Le ver plat commence à pondre dans les 3 semaines suivant l'éclosion (Kaneda et al., 1992). Le seuil de température pour la ponte se situe entre 15 °C et 18 °C, et pour les cocons et les stades juvéniles respectivement 10 °C et 11,7 °C (Kaneda et al., 1992). Le ver plat se reproduit normalement de manière sexuée, et ne semble pas se reproduire par scissiparité (Kaneda et al., 1990).

Biologie

Platydemus manokwari préfère des conditions humides et est incapable de survivre dans des habitats complètement secs ; une forte humidité et des précipitations suffisantes sont essentielles pour sa survie (Kaneda et al. 1990 ; Sugiura 2009). L'espèce est diurne, si les conditions d'humidité sont adéquates (Kaneda et al., 1990). La température semble influer sur le taux de prédation par l'espèce sur le terrain et en laboratoire, et également sa survie. Sugiura (2009) estime que 10 °C est le seuil de température pour l'implantation de *P. manokwari*, et spécule que les basses températures hivernales peuvent restreindre l'invasion et l'établissement de *P. manokwari* dans les pays tempérés.

Platydemus manokwari, comme un certain nombre d'autres Rhynchodeminae de la région Australie - Nouvelle-Guinée, semble être une espèce de montagne qui vit naturellement dans les zones alpines jusqu'à subalpines, les zones tempérées fraîches et chaudes et jusqu'aux climats tropicaux. À la station Pindaunde sur le mont Wilhelm, Nouvelle-Guinée, la température moyenne quotidienne est 11,6 °C, la température moyenne minimale 4 °C, le maximum absolu 16.7 °C, le minimum absolu de -0,8 °C, et les précipitations d'approximativement 3450 mm par an (Corlett 1984), bien qu'on suppose que le microclimat sur le sol de la forêt subalpine serait plus doux. Le climat à Pindaunde a été décrit comme « hivernal la nuit, (et) a des jours qui semblent appartenir à un printemps froid ou à l'automne » (McVean 1968). Dans le cas où *P. manokwari* s'échapperait de la serre et soit introduit dans des climats tempérés, le ver plat pourrait très bien survivre aux hivers et s'établir. Des hautes fréquences d'hivers chauds dans les zones tempérées peuvent également faciliter la mise en place du ver plat dans ces lieux (Sugiura, 2009). L'espèce a survécu dans

la serre à Caen, et nous estimons qu'elle pourrait également survivre à l'extérieur dans cette région, et plus facilement encore dans les parties de l'Europe plus au sud. Une évaluation de la distribution mondiale potentielle de *Platydemus manokwari*, sur la base de données éco-climatiques, n'a pas encore été entreprise.

Proies

Les mollusques terrestres constituent les proies principales sur lesquelles *Platydemus manokwari* a été observé se nourrir sur le terrain (Tableau 2) et dans des conditions de laboratoire et d'expérimentation (Tableau 3), bien que l'espèce se nourrisse également d'autres invertébrés vivant dans le sol, dont des annélides, arthropodes, némertes, et des vers plats (Tableaux 2 et 3). L'espèce ne semble pas être cannibale (Kaneda et al. 1990 ; Ohbayashi et al. 2005 ; Sugiura 2010).

Un certain nombre d'espèces de Plathelminthes terrestres peuvent, lorsque les conditions d'humidité sont favorables, chercher des proies au-dessus du sol. *Platydemus manokwari* a été observé se nourrissant de juvéniles et d'adultes d'escargots Partulidae à des hauteurs supérieures à un mètre dans les arbres, et en captivité l'espèce se nourrit de spécimens de *Partula* sp. et *Pythia* sp. (Eldredge & Smith, 1995 ; Hopper & Smith, 1992). Expérimentalement, il a été démontré que *P. manokwari* peut suivre des pistes d'escargots artificiellement créées sur le sol (Iwai et al. 2010) et en montant aux arbres, ce qui renforce l'hypothèse selon laquelle l'introduction de *P. manokwari* est une cause importante du déclin rapide ou de l'extinction d'escargots arboricoles indigènes et d'escargots terrestres dans les îles du Pacifique (Sugiura et Yamaura 2008).

Là où il y a suffisamment d'individus de *P. manokwari* suivant les signaux sensoriels de la même proie l'espèce peut submerger sa proie par une attaque grégaire en « bande organisée » (Mead, 1963 ;. Ohbayashi et al 2005 ; Sugiura 2010).

Waterhouse et Norris (1987) ont estimé que *P. manokwari* était un carnivore opportuniste et généralement non sélectif dans le choix de ses proies. Le succès de *Platydemus manokwari* comme agent de lutte biologique contre *Achatina fulica* peut être attribuée à sa polyphagie, sa résistance à la famine, sa capacité à survivre et se reproduire sur des proies de remplacement et son potentiel à se reproduire rapidement en synchronie avec les populations de proies (Winsor et al. 2004).

L'invasion d'un site par *Platydemus manokwari* peut avoir un impact direct et indirect sur les espèces terrestres et arboricoles indigènes et introduites et, dans une moindre mesure, sur la faune semi-aquatique d'invertébrés à mouvements lents.

Impacts

Du point de vue agricole *Platydemus manokwari* n'est pas un nuisible direct des plantes. En fait, il a été et sera probablement encore utilisé par les agriculteurs locaux et les organismes de protection des végétaux dans la région du Pacifique en tant qu'agent biologique dans le contrôle des foyers de l'escargot africain géant *Achatina fulica* (FAO-SAPA 2002 ; Winsor et al. 2004), bien que d'autres facteurs en dehors de la prédation par les vers plats puissent contribuer à la diminution des populations d'escargots ravageurs (Lydeard et al. 2004).

D'un point de vue environnemental, *P. manokwari* a manifestement eu un impact négatif grave sur la biodiversité des populations d'escargots indigènes dans la région du Pacifique (Cowie 2010), et là où il est délibérément ou accidentellement introduit il continuera de représenter une menace non seulement pour les mollusques indigènes, mais peut-être pour d'autres invertébrés lents du sol (Sugiura 2010). L'espèce peut aussi avoir un effet négatif indirect sur les espèces de vertébrés qui dépendent de ces invertébrés du sol.

Une évaluation du risque phytosanitaire et environnemental en accord avec les Normes Internationales pour les Mesures Phytosanitaires (IPPC 2004) pourrait être nécessaire pour *P. manokwari*, incluant : une évaluation de la probabilité de propagation directe du ver plat, considérée ici comme faible ; savoir si la population se reproduit activement et si elle est viable ; une évaluation des conséquences économiques, par exemple les menaces potentielles pour l'élevage commercial d'escargots ; les conséquences environnementales, par exemple les impacts négatifs sur la biodiversité des invertébrés du sol en France et ailleurs. Les points qui devraient également être considérés incluent l'ampleur de cette invasion, le fait de savoir si elle est limitée à la serre à Caen, la source probable primaire de l'incursion en cours, et la dispersion possible secondaire à travers des échanges de plantes entre les jardins botaniques et les centres de jardinage ou les supermarchés de plantes.

Options possibles pour le contrôle

Comme *Platydemus manokwari* n'est pas directement nuisible aux plantes, il ne figure pas dans les listes A1 ou A2 des organismes nuisibles recommandés pour réglementation

comme organismes de quarantaine de l'Organisation Européenne et Méditerranéenne pour la Protection des Plantes (OEPP) (EPPO 2013a, b), ni dans les listes du Réseau Européen d'Information sur les Espèces Invasives (EASIN 2014). En Europe les pays participants dans le réseau NOBANIS ont mis en place un système d'alerte précoce simple (Secrétariat de NOBANIS 2012). Quand un pays participant est informé que de nouvelles espèces exotiques ont été trouvées dans le pays, un avertissement est envoyé aux autres pays participants et affiché sur le site web NOBANIS. Cette alerte permet aux pays d'être alertés qu'une nouvelle espèce a été observée dans la région.

Selon les résultats d'une évaluation des risques environnementaux et des enquêtes connexes, il faudra probablement répondre aux menaces de *Platydemus manokwari* d'une manière similaire au ver plat invasif de Nouvelle-Zélande *Arthurdendyus triangulatus*. Cette espèce est maintenant l'objet d'une Norme OEPP sur les exigences d'importation (EPPO 2000a) et de contrôle, d'exclusion et de traitement de pépinière (EPPO 2000b) (Murchie 2010). Le problème avec *P. manokwari* est que, même si l'espèce est avant tout une menace pour l'environnement, on ne peut pas dire qu'elle « affecte les végétaux indirectement par les effets sur d'autres organismes ». Par conséquent, il est possible que la responsabilité de la gestion de cette espèce envahissante puisse échapper à la mission des organismes de réglementation de l'agriculture et de l'environnement. Cela pourrait retarder la gestion efficace de *P. manokwari*.

Contrôle chimique

Bien que toute une gamme de pesticides commerciaux ait été testée contre *Arthurdendyus triangulatus* seul le gamma-HCH (Lindane), un insecticide organochloré à large spectre, a montré un effet important mais a été considéré comme inapproprié pour le contrôle généralisé du ver plat (Cannon et al., 1999). Il peut y avoir une portée limitée pour l'utilisation de produits chimiques dans une approche intégrée pour le contrôle des vers plats exotiques envahissants, combinant méthodes chimiques, physiques et culturelles (Blackshaw 1996 ; Cannon et al. 1999).

Désinfection des plantes

Les traitements thermiques ou à l'eau chaude des plantes en container, qui tueraient les espèces exotiques envahissantes, ont été étudiés pour *Arthurdendyus triangulatus* et *Platydemus manokwari*. Des spécimens d'*A. triangulatus* ont été tués après immersion dans

un flacon pendant 5 minutes dans de l'eau à une température de 34 °C (Murchie & Moore, 1998). Cette méthode semblait très prometteuse (Cannon et al. 1999), mais ne semble pas avoir été largement utilisé. Le conseil actuel à certains composteurs amateurs qui ont des infestations de vers plats est de placer leur compost sous serre pour obtenir les températures aussi élevées que possible avant la diffusion du compost (Murchie, communication personnelle). Des expériences similaires ont été menées sur quatre taxons d'invertébrés du sol y compris *Platydemus manokwari*, en utilisant l'immersion dans l'eau chaude à des températures plus élevées (Sugiura 2008). Il a été constaté que l'exposition des animaux à l'eau chaude à des températures entre 43 °C et 50 °C pendant au moins 5 minutes a entraîné une mortalité de 100 % pour toutes les espèces testées. Dans les deux séries d'expériences les vers plats ont été testés dans des flacons en plastique. La capacité de traitement de l'eau chaude pour tuer les animaux dans la masse des sols en pots n'a pas été examinée. En fonction de sa capacité de porosité et de sa contenance en eau le sol peut agir comme un tampon thermique. Une méthode plus prometteuse de traitement à l'eau chaude est la méthode d'arrosage de Tsang (2001) visant à assainir les plantes en pot des nématodes fouisseurs et d'autres espèces de ravageurs potentiels. Le traitement dans lequel on arrose les plantes en pot avec de l'eau chaude à 50 °C pendant 15-20 minutes est plus efficace pour tuer les nématodes fouisseurs que l'immersion des plantes en pot dans l'eau chaude avec le même régime température / temps. Sur la base des données de Sugiura (2008) ce régime température/temps de circulation d'eau chaude tuerait *Platydemus manokwari*. L'appareil d'arrosage pourrait se prêter à un développement et une utilisation commerciale.

Lutte biologique

Pour l'instant il n'y a pas de méthodes de lutte biologique spécifique connue contre *Platydemus manokwari*. Les vers plats terrestres sont considérés comme des prédateurs de haut niveau dans l'écosystème du sol (Sluys 1999). Rien ne semble être connu sur les ennemis naturels de *Platydemus manokwari*, mais on connaît des exemples de prédation sur d'autres espèces de Plathelminthes terrestres par la faune du sol et associée au sol, la plupart du temps dans des conditions de laboratoire. Ces exemples incluent une instance de prédation de l'espèce néotropicale *Obama trigueira* (EM Froehlich, 1955) par *Enterosyringia pseudorhynchodemus* (Riester, 1938) (Froehlich 1956) et la prédation de cinq espèces de Plathelminthes terrestres par *P. manokwari* (Ohbayashi et al. 2005). Une observation de hasard sur le terrain a conduit à des résultats de laboratoire montrant qu'*Arthurdendyus*

triangulatus est mangé par les larves et les adultes d'espèces de coléoptères carabidés et staphylinidés (Gibson et al. 1997).

Dans une série d'essais de Lemos et al. (2012), il a été montré que le mollusque néotropical carnivore indigène *Rectartemon depressus* (Heynemann, 1868) peut être prédateur avec succès sur des spécimens d'au moins 10 espèces de vers plats terrestres Geoplanidae ainsi que cinq espèces non décrites de *Geoplana*, et sur l'espèce introduite *Bipalium kewense*. On ne sait pas encore si d'autres espèces de mollusques carnivores peuvent être prédatrices de vers plats. *Platydemus manokwari* est prédateur d'au moins deux espèces de mollusques carnivores observées sur le terrain (Ohbayashi et al. 2005) : *Euglandina rosea* (de Férussac, 1821) et *Gonaxis quadrilateralis* (Preston, 1901) ; ces deux espèces de mollusques ont été introduites dans une tentative de contrôler l'escargot géant africain *Achatina fulica* dans la région Pacifique (Davies et Butler, 1964 ; Lydeard et al. 2004).

Platydemus manokwari a un goût astringent des plus désagréables (obs. pers. LW), comme cela a été constaté pour d'autres espèces (Dendy 1891). Bellwood (communication personnelle à LW, 13.11.1997), dans son jardin urbain privé, a noté que des poules naines domestiques qui remarquaient *P. manokwari* sur une souche retournée becquetaient les vers, les prenaient dans leur bouche, puis les rejetaient tout de suite. Quand plus tard des *P. manokwari* ont été remarqués par les poules, elles refusèrent de picorer les vers plats. Ceci est similaire au comportement des oiseaux domestiques auxquels on a proposé *Caenoplana spenceri* (Dendy 1891). La prédation des vers plats par des espèces indigènes d'oiseaux n'a pas été signalée.

La prédation des vers plats terrestres par l'herpétofaune a également été étudiée. Le ver plat *Bipalium adventitium* Hyman, 1943, envahissant en Amérique du Nord, a été proposé par Ducey et al. (1999) à six espèces de salamandres et deux espèces de serpents ; aucune des espèces de l'herpétofaune testées n'a traité *Bipalium adventitium* comme proie potentielle.

Le parasitisme de *P. manokwari* par des nématodes, grégarines ou diptères Mycetophilidae, décrit chez d'autres espèces de Plathelminthes terrestres (Graff 1899 ; Hickman, 1964), n'a jamais été observé.

Lemos et al. (2012) préconisent des essais expérimentaux supplémentaires sur d'autres invertébrés et vertébrés prédateurs potentiels de vers plats, dans le but de mieux comprendre les relations prédateur-proie. Conscients des risques associés à la lutte biologique, ils considèrent que l'utilisation d'espèces non-indigènes doit être évitée, et si nécessaire, doit être basée sur des tests détaillés avant et après le relâchage.

Conclusion

Les impacts environnementaux négatifs graves de *Platydemus manokwari* sur la biodiversité des escargots terrestres indigènes de la région Indo-Pacifique sont bien documentés. Les risques posés par l'incursion de cette espèce en France n'ont pas encore été évalués. L'Union européenne a récemment émis une nouvelle proposition législative visant à prévenir et à gérer le danger toujours plus grand que représentent les espèces exotiques envahissantes contre la biodiversité (Commission européenne 2013). La proposition se concentre sur une liste d'espèces exotiques envahissantes préoccupante pour l'Europe, qui sera établie avec les États membres en utilisant des évaluations des risques et des preuves scientifiques. Que *Platydemus manokwari* soit ou non inclus dans cette liste reste à voir.

Remerciements

Les auteurs tiennent à remercier Gérard Peaucellier et Mme J. Vivant pour des informations sur les vers plats terrestres en France, David Bellwood à Townsville, dans le Queensland, pour ses observations sur la réponse alimentaire des poules domestiques à *P. manokwari*, Gilianne Brodie (School of Biological and Chemical Sciences, University of the South Pacific, Fidji) pour ses articles sur l'apparition de *Platydemus manokwari* à Rotuma, Archie Murchie (Agri-Food and Biosciences Institute, Belfast, Irlande du Nord) pour informations et conseils sur les pratiques actuelles d'assainissement des plantes, et les normes et législation pour l'espèce exotique envahissante *Arthurdendyus triangulatus,* Nicolas Puillandre (MNHN, Paris) pour l'aide avec les séquences, Pierre Lozouet (MNHN, Paris) pour l'identification de l'escargot. Nous sommes particulièrement reconnaissants à Damien Loisel et David Philippart (FREDON Basse-Normandie, France) et Martine Aires (DRAAF Basse-Normandie, France) pour la collecte des échantillons et le soutien administratif et à Damien L'Hours et Nelly Hubert (Ville de Caen, France) pour avoir permis la collecte dans la serre à Caen et fourni un soutien administratif. Hugh Jones, Ana M. Leal-Zanchet, Fernando Carbayo et Maria Riutort sont remerciés pour leurs commentaires constructifs sur une version antérieure de cet article.

Tableau 1. Mentions de *Platydemus manokwari*

Platydemus manokwari a été signalé dans plus de 15 territoires différents, en Asie et Océanie ; notre signalement en France est le premier pour l'Europe

Territoire	Année de découverte	Localité, commentaires	Référence
**Irian Jaya**	1962	Agricultural Research Station, Manokwari	de Beauchamp 1962
**Nouvelle Guinée**	1969	Mt Wilhelm, Pindaude Station	de Beauchamp 1972
	1973	Kainantu, 45 km SE de Goroka	LW identification pour C. Vaucher *in litt* 4.iii.1982
**Australie**	1976	Queensland : Lockhart River, Weipa, Atherton Tablelands, Cairns, Mission Beach, Cardwell, Crystal Creek ; Bluewater, Townsville.	LW 1985 comm. pers.. in Waterhouse & Norris 1987 ; Winsor 1990, 1999
	2002	Northern Territory : Anula	LW identification pour C. Glasby *in litt* 23.x.2002
	2009	Queensland : Bowen ; Airlie Beach	LW collection
**Guam, Iles Marianne du Nord**	1977-1984	Guam, Saipan, Tinian	Hopper & Smith 1992 ; Eldredge & Smith 1995
	1988	Rota	Bauman 1996
	1992	Aguijan, identifié comme "Microplaninae sp."	Eldredge & Smith 1995 ; Kawakatsu & Ogren 1994
**Philippines**	1981	Bugsuk, introduction délibérée	Muniappan et al. 1986 ; Waterhouse & Norris 1987
	1985	Manilla, urbain	Waterhouse & Norris 1987
**Japon**	1984	Yokohama, introduction délibérée depuis Saipan	Eldredge & Smith 1995
	1990-1991	Ile Okinawa et autres Iles Ryukyu	Eldredge & Smith 1995 ; Kawakatsu et al. 1993
	1995	Ile Ogasawara (Bonin)	Kawakatsu et al. 1999
**Maldives**	1985	Fua Mulaku, introduction délibérée depuis Bugsuk et Saipan ; Addu Atoll ; Male Atoll	Eldredge & Smith 1995 ; Muniappan 1987
**Palau**	1991	Ile Koror	Eldredge & Smith 1995
	1992	Ile Ulong	Eldredge & Smith 1995
**Hawaii**	1992	Oahu	Eldredge & Smith 1995
**États fédérés de Micronésie**		Ile Pohnpei Ponape	Eldredge & Smith 1995
**Polynésie Française**	1997	Ile Mangareva, route vers Mt Mokoto	LW identification pour J. Starmer *in litt* 21.iii.1998 ; Purea et al. 1998
	2009	Ile Moorea	Lovenburg 2009
**Samoa**	1998	Alafua et Upolu	Cowie & Robinson 2003 ; Purea et al. 1998
**Tonga**			FAO-SAPA 2002
**Vanuatu**			FAO-SAPA 2002
**Fidji**	2012	Rotuma	Brodie et al. 2012, 2013
**France**	2013	Caen, Normandie	Cet article

Tableau 2. Espèces rapportées comme proies de *Platydemus manokwari*, sur le terrain

On a rapporté que *Platydemus manokwari* se nourrit principalement de mollusques gastropodes, et aussi de vers de terre, insectes et némertes.

Espèce	Localité	Référence
**Mollusca : Gastropoda**		
Achatina fulica Bowdich	Manokwari, Irian Jaya Agricultural Research Station	Hopper & Smith 1992 ; Kawakatsu et al. 1992, 1993 ; Mead 1963, 1979 ; Muniappan et al. 1986 ; Raut & Barker 2002 ; Schreurs 1963 ; Sugiura 2010 ; van Driest 1968 ; Waterhouse & Norris 1987 ; Winsor 1999
Euglandina rosea (de Férussac)	Okamura, Ile Chichijima, Ile Ogasawara, Japon	Ohbayashi et al. 2005
Partulidae : juvenile *Partula*, Streptaxidae : *Gonaxis quadrilateralis* (Preston),	Guam	Hopper & Smith 1992
Planorbidae : *Physastra* sp.	north Queensland, Australia urbain	Waterhouse & Norris 1987
“Limaces” probablement Vaginulidae	Nouvelle Guinée	Winsor et al. 2004
“Escargot prédateur introduit” probablement Streptotaxidae : *Gonaxis quadrilateralis, Incilaria* sp. cadavre	Nouvelle Guinée, Chou-zan, Ile Chichijima, Iles Ogasawara, Japon	Ohbayashi et al. 2005
**Annelida : Oligochaeta**		
Ver de terre pheretimoidé	north Queensland, Australie urbain	Waterhouse & Norris 1987
Haplotaxida spp. cadavre	Komagari, Ile Chichijima, Iles Ogasawara, Japon	Ohbayashi et al. 2005
**Arthropoda : Insecta**		
Blattellidae : *Calolampra* sp.	north Queensland, Australie urbain	Waterhouse & Norris 1987
**Nemertea : Enopla**		
Geonemertes pelaensis Semper	Chou-zan, Ile Chichijima, Iles Ogasawara, Japon	Ohbayashi et al. 2005
**Vertebrata : Amphibia**	north Queensland, Australie urbain	Waterhouse & Norris 1987
Litoria coerulea (White) cadavre		

Tableau 3. Espèces signalées comme proies de *Platydemus manokwari*, en conditions de laboratoire

Platydemus manokwari est capable de se nourrir d'un grand nombre d'espèces de mollusques, de némertes, de vers de terre et de cloportes, et d'autres espèces de Plathelminthes terrestres. Toutes les mentions de proies sont des adultes.

Espèce	Référence
**Mollusca : Gastropoda**	
Partula sp.	Hopper 1990
Bradybaenidae : *Acusta despecta sieboldiana* (Pfeiffer), *Bradybaena similaris* (de Férussac), *Euhadra amaliae callizona* (Crosse), *Euhadra peliomphala* (Pfeiffer), *Euhadra quaesita* (Deshayes), *Trishoplita conospira* (Pfeiffer) ; Camaenidae : *Satsuma japonica* (Pfeiffer) ; Clausiliidae : *Euphaedusa tau* (Boettger), *Pinguiphaedusa hakonensis* (Pilsbry), *Zaptychopsis buschi* (Pfeiffer) ; Discidae : *Discus pauper* (Gould) ; Helicarionidae : *Helicarion* sp. ; Limacidae : *Lehmannia marginata* (Müller) ; Ellobiidae : *Pythia scarabaeus* Linnaeus ; Zonitidae : *Zonitides arboreus* (Say) ; Achatinidae : *Achatina fulica*	Kaneda et al. 1990, 1992
Achatina fulica, *Limax marginatus* (Müller), *Deroceras laeve* (Müller), *Euglandina rosea*, *Bradybaena similaris* (de Férussac), *Acusta despecta sieboldiana* (Pfeiffer)	Ohbayashi et al. 2005
Acusta despecta (Pfeiffer)	Sugiura 2010
Helicidae : *Eobania vermiculata* Müller	Cet article
**Platyhelminthes : Tricladida**	
Australopacifica sp., *Bipalium kewense*, *Bipalium* sp., *Platydemus* sp. 1 ; *P.* sp. 2	Ohbayashi et al. 2005
**Nemertea : Enopla**	
Geonemertes pelaensis Semper	Ohbayashi et al. 2005
**Annelida : Oligochaeta**	
Eisenia foetida Savigny	Sugiura 2010
**Arthropoda : Crustacea**	
Armadillidium vulgare Latreille	Sugiura 2010

Références

Alford DV, Lole MJ, and Emmett BJ. 1996. Alien terrestrial planarians in England and Wales, and implications for horticultural trade. *Brighton crop protection conference: pests & diseases - 1996: volume 3: Proceedings of an international conference, Brighton, UK, 18-21 November 1996*, 1083-1088.

Alvarez-Presas M, Mateos E, Vila-Farré M, Sluys R, and Riutort M. 2012. Evidence for the persistence of the land planarian species *Microplana terrestris* (Müller, 1774) (Platyhelminthes, Tricladida) in microrefugia during the Last Glacial Maximum in the northern section of the Iberian Peninsula. *Molecular Phylogenetics and Evolution* 64:491-499.

Bauman S. 1996. Diversity and decline of land snails on Rota, Mariana Islands. *American Malacological Bulletin* 12:13-27.

Blackshaw RP. 1996. Control options for the New Zealand flatworm. Brighton Crop Protection Conference – Pests and Diseases. Volume III, pp. 1089-1094.

Boag B, Palmer LF, Neilson R, and Chambers SJ. 1994. Distribution and prevalence of the predatory planarian *Artioposthia triangulata* (Dendy) (Tricladida: Terricola) in Scotland. *Annals of Applied Biology* 124:165-171.

Boag B, and Yeates GW. 2001. The potential impact of the New Zealand flatworm, a predator of earthworms, in Western Europe. *Ecological Applications* 11:1276-1286.

Breugelmans K, Quintana Cardona J, Artois T, Jordaens K, and Backeljau T. 2012. First report of the exotic blue land planarian, *Caenoplana coerulea* (Platyhelminthes, Geoplanidae), on Menorca (Balearic Islands, Spain). *Zookeys* 199:91-105.

Brodie G, Barker GM, Stevens F, and Fiu M. 2013. Preliminary re-survey of the land snail fauna of Rotuma: conservation and biosecurity implications. *Pacific Conservation Biology* 20:in press.

Brodie G, Stevens F, and Barker G. 2012. Report on a preliminary survey of the land snail fauna of Rotuma. http://www.rotuma.net/os/Publications/land_snail_survey.pdf

Cannon RJC, Baker RHA, Taylor MC, and Moore JP. 1999. A review of the status of the New Zealand flatworm in the UK. *Annals of Applied Biology* 135:597-614.

Christensen OM, and Mather JG. 1998. The 'New Zealand flatworm', *Artioposthia triangulata*, in Europe: the Faroese situation. *Pedobiologia* 42 532-540.

Convention on Biological Diversity. 2009. What are Invasive Alien Species? http://www.cbd.int/idb/2009/about/what/default.shtml (accessed 06-Fev-2014).

Corlett RT. 1984. Human impact on the subalpine vegetation of Mt Wilhelm, Papua New Guinea. *Journal of Ecology* 72:841-854.

Cowie RH, and Robinson AC. 2003. The decline of native Pacific island faunas: changes in status of the land snails of Samoa through the 20th century. *Biological Conservation* 110:55-65.

Cowie RHC. 2010. *Platydemus manokwari* (flatworm) profile. In: Global Invasive Species Database. http://www.issg.org/database/welcome. Accessed 09/11/2013.

Davis CJ, and Butler GD. 1964. Introduced enemies of the Giant African snail, *Achatina fulica* Bowdich, in Hawaii (Pulmonata: Achatinidae). *Proceedings of the Hawaiian Entomological Society* 18: 377-390.

de Beauchamp P. 1962. *Platydemus manokwari* n. sp., planaire terrestre de la Nouvelle-Guinée Hollandaise. *Bulletin de la Société Zoologique de France* 87:609-615.

de Beauchamp P. 1972. Planaires terrestres de Nouvelle-Guinée. *Cahiers du Pacifique* 16:181-192.

Dendy A. 1891. The Victorian land planarians. *Transactions of the Royal Society of Victoria 2 (for 1890):* 65-80.

Ducey PK, Messere M, Lapoint K, and Noce S. 1999. Lumbricid prey and potential herpetofaunal predators of the invading terrestrial flatworm *Bipalium adventitium* (Turbellaria: Tricladida: Terricola). *American Midland Naturalist* 141: 305-314.

Ducey PK. McCormick M, and Davidson E. 2007. Natural history observations on *Bipalium* cf. *vagum* Jones and Sterrer (Platyhelminthes: Tricladida), a terrestrial broadhead planarian new to North America. *Southeastern Naturalist* 6: 449-460.

Ducey PK, West L-J, Shaw G, and De Lisle J. 2005. Reproductive ecology and evolution in the invasive terrestrial planarian *Bipalium adventitium* across North America. *Pedobiologia* 49: 367-377.

Eldredge LG, and Smith BD. 1995. Triclad flatworm tours the Pacific. *Aliens* 2:11.

EASIN (European Alien Species Information Network). 2014 Terrestrial alien species in europe. http://easin.jrc.ec.europa.eu

EPPO (European and Mediterranean Plant Protection Organization). 2000a. EPPO Standards. Guidelines on *Arthurdendyus triangulatus*. Import requirements concerning *Arthurdendyus triangulatus*. http://archives.eppo.int/EPPOStandards/PM1 GENERAL/pm1-03-e.doc

EPPO (European andMediterranean Plant Protection Organization). 2000b. Guidelines on *Arthurdendyus triangulatus*. Nursery inspection, exclusion and treatment for *Arthurdendyus triangulatus*. http://archives.eppo.int/EPPOStandards/PM1_GENERAL/pm1-04-e.doc

EPPO (European and Mediterranean Plant Protection Organization). 2013a. EPPO A1 List of pests recommended for regulation as quarantine pests (version 2013-09). http://www.eppo.int/QUARANTINE/listA1.htm

EPPO (European and Mediterranean Plant Protection Organization). 2013b. EPPO A2 List of pests recommended for regulation as quarantine pests (version 2013-09). http://www.eppo.int/QUARANTINE/listA2.htm

European Commission. 2013 Press release 09/09/2013. Environment: New EU Action to protect biodiversity against problematic invasive species. http://europa.eu/rapid/press-release_IP-13-818_en.htm

Filella-Subirá E (1983) Nota sobre la presència de la planària terrestre *Bipalium kewense* Mosoley [sic], 1878 a Catalunya. Butlleti de la Institució Catalana d'Història Natural 49: 151.

FAO-SAPA (Food and Agriculture Organization Sub-regional officer for the Pacific Islands). 2002. Bio-control: flatworms and nemertean worms collected and identified from Cook Islands, Niue, Tonga and Vanuatu. *SAPA Newsletter* 6:3.

Froehlich CG. 1956. On the biology of land planarians. *Boletim da Faculdade de filosofia, ciéncias e letras, Universidade de São Paulo* 20: 263-272.

Gasiglia S. 2013. À Cagnes, alerte au ver plat mangeur de lombrics. Nice-Matin. Nice. June 2, 2013, page 8.

Gibson PH, Cosens D, and Buchanan K. 1997. A chance field observation and pilot laboratory studies of predation of the New Zealand flatworm by the larvae and adults of carabid and staphylinid beetles. *Annals of Applied Biology* 130: 581-585.

Graff L von. 1899. Monographie der Turbellaria. II Tricladida Terricola (Landplanarien). Text & Atlas. Wilhelm Engelmann: Leipzig.

Groult J-M, and Boucourt F. 2014. Les vers exotiques menacent nos lombrics. Mon Jardin & Ma Maison. N° 648, January 2014, pages 100-101.

Guyon J. 2014. Le ver tueur de lombrics est entré dans Paris. Le Parisien. Paris. January 5, 2014, page 14. http://www.leparisien.fr/espace-premium/paris-75/le-ver-tueur-de-lombrics-est-entre-dans-paris-05-01-2014-3462717.php

Heyligen J. 2013. Le tueur de lombrics repéré dans le département. Le Parisien, édition Essonne. Paris. October 4, 2013. http://www.leparisien.fr/espace-premium/essonne-91/le-tueur-de-lombrics-repere-dans-le-departement-04-10-2013-3194081.php

Hickman VV. 1964. On *Planarivora insignis* Gen. et sp.n. (Diptera; Mycetophilidae), whose larval stages are parasitic in land planarians. *Papers and Proceedings of the Royal Society of Tasmania* 99: 1-8.

Hopper D. 1990. Threats to *Partula* on Guam, Mariana Islands. *Tentacle* 1:3-4.

Hopper DR, and Smith BD. 1992. Status of tree snails (Gastropoda: Partulidae) on Guam, with a resurvey of sites studied by H. E. Crampton in 1920. *Pacific Science* 46:77-85.

Hyman LH. 1954. Some land planarians of the United States and Europe, with remarks on nomenclature. *American Museum Novitates* No. 1667: 1-21.

International Commission on Zoological Nomenclature. 1999. *International Code of Zoological Nomenclature*: The International Trust for Zoological Nomenclature.

IPPC (International Plant Protection Convention). 2004. International Standards for Phytosanitary Measures: Pest risk analysis for quarantine pests, including analysis of environmental risks and living modified organism. International Standards for Phytosanitary Measures No. 11, 117-138.

Iwai N, Sugiura S, and Chiba S. 2010. Prey-tracking behavior in the invasive terrestrial planarian *Platydemus manokwari* (Platyhelminthes, Tricladida). *Naturwissenschaften* 97:997-1002.

Jones HD. 1998. The African and European land planarians faunas, with an identification guide for field workers in Europe. *Pedobiologia* 42:477-489.

Jones HD. 2005. Identification: British land flatworms. *British Wildlife* 16:189-194.

Jones HD, Santoro G, Boag B, and Neilson ROY. 2001. The diversity of earthworms in 200 Scottish fields and the possible effect of New Zealand land flatworms (*Arthurdendyus triangulatus*) on earthworm populations. *Annals of Applied Biology* 139:75-92.

Justine J-L. 2013. Blog: Plathelminthes terrestres invasifs. https://sites.google.com/site/jljjustine/plathelminthe-terrestre-invasif or http://bit.ly/Plathelminthe

Kaneda M, Kitagawa K, and Ichinohe F. 1990. Laboratory rearing method and biology of *Platydemus manokwari* de Beauchamp (Tricladida: Terricola: Rhynchodemidae). *Applied Entomology and Zoology* 25:524-528.

Kaneda M, Kitagawa K, Nagai H, and Ichinohe F. 1992. The effects of temperature and prey species on the development and fecundity of *Platydemus manokwari* de Beauchamp (Tricladida: Terricola: Rhynchodemidae). *Research Bulletin of Plant Protection Series of Japan* 28:7-11.

Kawakatsu M, and Ogren RE. 1994. A preliminary report on land planarians from the Northern Mariana Islands (Turbellaria, Tricladida, Terricola). *Natural History Research Special Issue* 1:107-112.

Kawakatsu M, Ogren RE, Froehlich EM, Takai M, and Sasaki G-Y. 2002. Additions and corrections of the previous land planarians indicies of the world (Turbellaria, Seriata, Tricladida, Terricola). Additions and corrections of the previous land planarians indicies of the world – 10. *Bulletin of the Fuji Women's College* No. 40:162-177.

Kawakatsu M, Ogren RE, and Muniappan R. 1992. Redescription of *Platydemus manokwari* de Beauchamp, 1962 (Turbellaria: Tricladida: Terricola), from Guam and the Philippines. *Proceedings of the Japanese Society of Systematic Zoology* 47:11-25.

Kawakatsu M, Oki I, Tamura S, Itô, Nagai Y, Ogura K, Shimabukuro S, Ichinohe F, Katsumata H, and Kaneda M. 1993. An extensive occurrence of a land planarian, *Platydemus manokwari* de Beauchamp, 1962, in the Ryûkuû Islands, Japan (Turbellaria, Tricladida, Terricola). *Biology of Inland Waters* 8:5-14.

Kawakatsu M, Okochi K, Sato H, Okochi K, Ohbayashi T, Kitagawa K, and Totani K. 1999. A preliminary report on land planarians (Turbellaria, Seriata, Tricladida, Terricola) and land nemertine (Enopla, Hoplonemertea, Monostylifera) from the Ogasawara Islands. *Occasional Publications, Biological Laboratory of Fuji Women's College, Sapporo (Hokkaidô)* 32:1-8.

Lemos VS, Canello R, and Leal-Zanchet AM. 2012. Carnivore mollusks as natural enemies of invasive land flatworms. *Annals of Applied Biology* 161:127-131.

Littlewood DTJ, Rohde K, and Clough KA. 1997. Parasite speciation within or between host species? - Phylogenetic evidence from site-specific polystome monogeneans. *International Journal for Parasitology* 27:1289-1297.

Lovenburg V. 2009. Terrestrial gastropod distributional factors: native and nonnative forests, elevation and predation on Mo'orea, French Polynesia. *UC Berkeley: UCB Moorea Class: Biology and Geomorphology of Tropical Islands.* http://www.escholarship.org/uc/item/73t4j6xs

Lowe S, Browne M, Boudjelas S, and De Poorter M. 2000. 100 of the World's Worst Invasive Alien Species. A selection from the Global Invasive Species Database. *Published by The Invasive Species Specialist Group (ISSG) a specialist group of the Species Survival Commission (SSC) of the World Conservation*

Union (IUCN), 12pp. First published as special lift-out in Aliens 12, December 2000. Updated and reprinted version: November 2004.

Lydeard C, Cowie RH, Ponder WF, Bogan AE, Bouchet P, Clark SA, Cummings KS, Frest TJ, Gargominy O, Herbert DG et al.2004. The global decline of nonmarine mollusks. *Bioscience* 54:321-330.

Mateos, E., Tudo, A., Alvarez-Presas, M. & Riutort, M. 2013: Planàries terrestres exòtiques a la Garrotxa. *Annals de la Delegació de la Garrotxa de la Institució Catalana d'Història Natural 6 :* 67-73.

McVean DN. 1968. A year of weather records at 3480m on Mt Wilhelm, New Guinea. *Weather* 23:377-381.

Mead AR. 1963. A flatworm predator of the giant African snail *Achatina fulica* in Hawaii. *Malacologia* 1:305-311.

Mead AR. 1979. Economic malacology with particular reference to *Achatina fulica*. In: Fretter V, and Peaks J, eds. *Pulmonates, Vol 2B*. London: Academic Press.

Moore JP, Dynes C, and Murchie AK. 1998. Status and public perception of the "New Zealand flatworm", *Artioposthia triangulata* (Dendy), in Northern Ireland. *Pedobiologia* 42:563-571.

Muniappan R. 1987. Biological control of the giant African snail, *Achatina fulica* Bowdich, in the Maldives. *FAO Plant Protection Bulletin* 35:127-133.

Muniappan R, Duhamel G, Santiago RM, and Acay DR. 1986. Giant African snail control in Bugsuk island, Philippines, by *Platydemus manokwari*. *Oléagineux* 41:183-186.

Murchie AK. 2010. Between two stools: dealing with the problem of the New Zealand flatworm. *Aspects of Applied Biology* 104:73-78.

Murchie AK, and Gordon AW. 2013. The impact of the "New Zealand flatworm", *Arthurdendyus triangulatus*, on earthworm populations in the field. *Biological Invasions* 15:569-586.

Murchie AK, and Moore JP. 1998. Hot-water treatment to prevent transference of the “New Zealand flatworm”, *Artioposthia triangulata*. *Pedobiologia* 42:572.

Ogren RE, Kawakatsu M, and Froehlich EM. 1997. Additions and corrections of the previous land planarian indicies of the world (Turbellaria: Tricladida: Terricola). Addendum IV. Geographic locus index: Bipaliidae; Rhynchodemidae (Rhynchodeminae; Microplaninae); Geoplanidae (Geoplaninae; Caenoplaninae; Pelmatoplaninae). *Bulletin of the Fuji Women’s College (Series 2)* 35:63-103.

Ohbayashi T, Okochi I, Sato H, and Ono T. 2005. Food habit of *Platydemus manokwari* De Beauchamp, 1962 (Tricladida: Terricola: Rhynchodemidae), known as a predatory flkatworm of land snails in the Ogasawara Islands, Japan. *Japanese Journal of Applied Entomology and Zoology* 40:609-614.

Placé J-M. 2013. Apparition d'une espèce de plathelminthe dans les sols de l'Essonne. Question écrite n° 09593 de M. Jean-Vincent Placé, publiée dans le Journal Officiel du Sénat du 05/12/2013 - page 3466. http://www.senat.fr/questions/base/2013/qSEQ131209593.html.

Purea M, Matalavea S, Bourke T, and Hunter D. 1998. *Platydemus manokwari* de Beauchamp, a flatworm predator of the giant African snail (*Achatina fulica* Bowdich) recorded in Samoa. *Journal of South Pacific Agriculture* 5:71-72.

Raut SK, and Barker GM. 2002. *Achatina fulica* Bowdich and other Achatinidae as pests in tropical agriculture. In: Barker GM, ed. *Molluscs as Crop Pests*. Wallingford, U.K. : CAB International, 54-114.

Remy P. 1942 Quelques Arthropodes intéressants des serres du Parc de la Tête d'Or. *Bulletin Mensuel de la Société Linnéenne de Lyon* 9:140-142.

Santoro G, and Jones HD. 2001. Comparison of the earthworm population of a garden infested with the Australian land flatworm (*Australoplana sanguinea alba*) with that of a non-infested garden. *Pedobiologia* 45:313-328.

Schreurs J. 1963. Investigations on the biology, ecology and control of the giant African snail in west New Guinea. Unpublished report, Manokwari Agricultural Research Station.

Secretariat of NOBANIS. 2012. *Riskmapping for 100 nonnative species in Europe.* http://www.nobanis.org/files/Riskmapping_report.pdf . Copenhagen: NOBANIS – European Network on Invasive Alien Species.

Simberloff D. 2014. Biological invasions: What's worth fighting and what can be won? *Ecological Engineering*. doi: 10.1016/j.ecoleng.2013.08.004

Sluys R. 1999. Global diversity of land planarians (Platyhelminthes, Tricladida, Terricola): a new indicator-taxon in biodiversity and conservation studies. *Biodiversity and Conservation* 8: 1663-1681.

Sugiura S. 2008. Hot water tolerance of soil animals : utility of hot water immersion in preventing invasions of alien soil animals. *Applied Entomology and Zoology* 43:207-212.

Sugiura S. 2009. Seasonal fluctuation of invasive flatworm predation pressure on land snails: Implications for the range expansion and impacts of invasive species. *Biological Conservation* 142:3013-3019.

Sugiura S. 2010. Prey preference and gregarious attacks by the invasive flatworm *Platydemus manokwari*. *Biological Invasions* 12:1499-1507.

Sugiura S, and Yamaura Y. 2008 Potential impacts of the invasive flatworm *Platydemus manokwari* on arboreal snails. *Biological Invasions* 11:737-742.

Tamura K, Peterson D, Peterson N, Stecher G, Nei M, and Kumar S. 2011. MEGA5: Molecular evolutionary genetics analysis using maximum likelihood, evolutionary distance, and maximum parsimony methods. *Molecular Biology and Evolution* 28:2731-2739.

Tsang MMC, Hara AH, and Sipes BS. 2001. A hot water drenching system for disinfesting roots and media of potted plants of the burrowing nematodes. *Applied Engineering in Agriculture* 17:533-538.

van Driest JT. 1968. Correspondentieblad. *Nederlandse Malacologie Vereniging* 127:1361-1362.

Vivant J. 2005. *Bipalium kewense* Moseley, ver tropical terricole, existe à Orthez (Pyr. atl.). *Bulletin de la Société Mycologique Landaise* 30: 46-48.

Waterhouse DE, and Norris KR. 1987. *Biological Control: Pacific prospects*. Canberra: Australian Centre for International Agricultural Research.

Winsor L. 1990. Taxonomic studies on free-living flatworms (Turbellaria: Platyhelminthes) of the Australian Zoogeographic Region. Chapter 4: Taxonomy and biology of a molluscivorous terrestrial flatworm *Platydemus manokwari* Beauchamp, 1962. MSc thesis. James Cook University: Townsville.

Winsor L. 1999. The New Guinea flatworm – *Platydemus manokwari*: predator of land snails. Terrestrial flatworms Infosheet No. 6. James Cook University. Townsville.

Winsor L, Johns PM, and Barker GM. 2004. Terrestrial planarians (Platyhelminthes: Tricladida: Terricola) predaceous on terrestrial gastropods. In: Barker GM, ed. *Natural enemies of terrestrial molluscs*. Oxfordshire, UK: CAB International, 227-278.

Figure 1. *Platydemus manokwari* de Beauchamp, 1963. Spécimen dans une serre à Caen, France. Vue dorsale : remarquer la ligne longitudinale médiane.

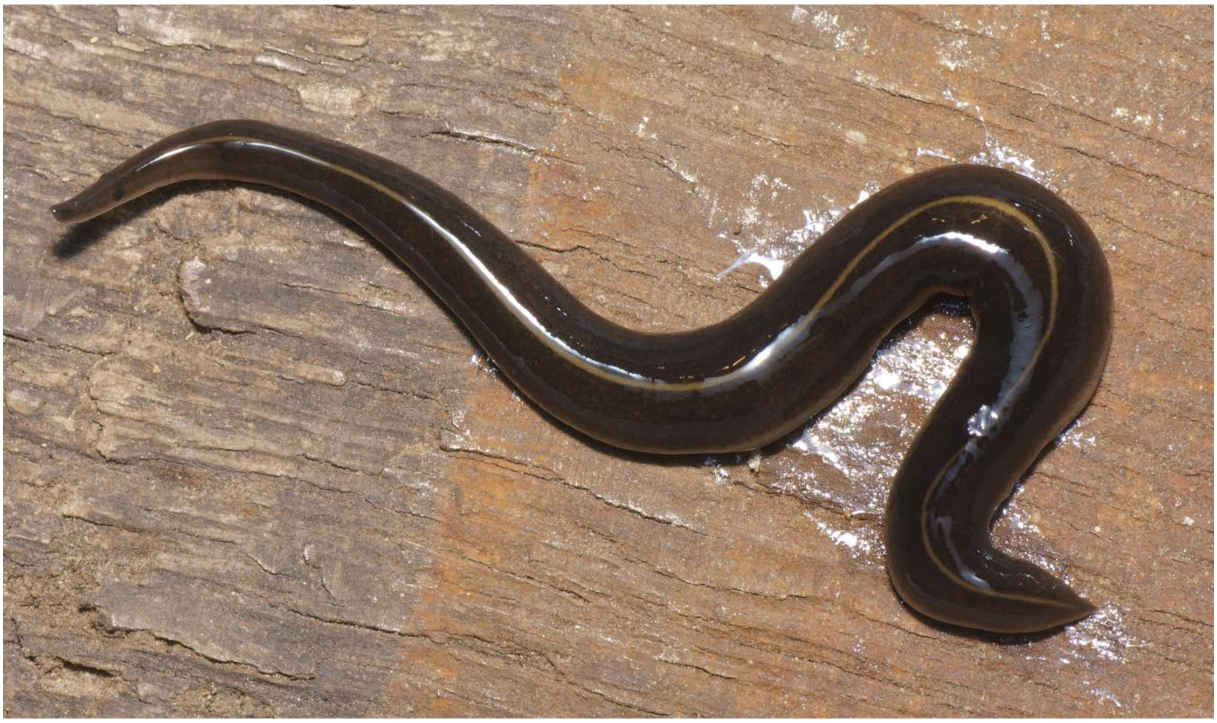

Figure 2. *Platydemus manokwari* de Beauchamp, 1963. Détail de la tête, montrant un des yeux, légèrement proéminent.

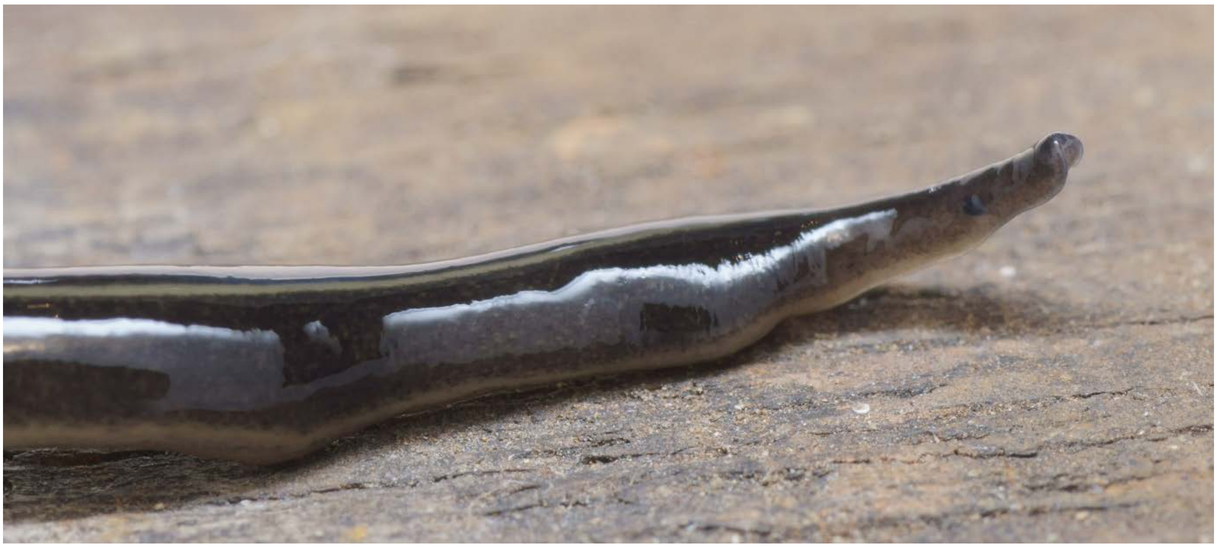

Figure 3. ***Platydemus manokwari*** **de Beauchamp, 1963.** Détail du corps, vue dorsale, montrant une ligne longitudinale médiane crème pale sur un fond couleur olive noire.

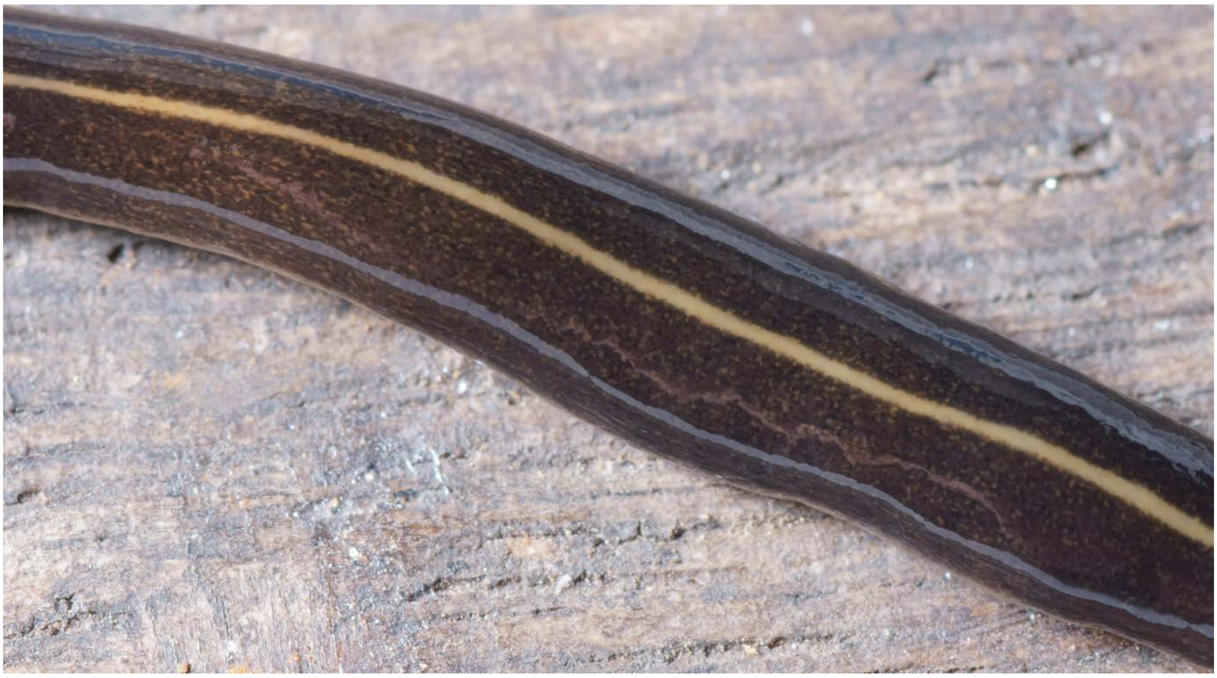

Figure 4. ***Platydemus manokwari*** **de Beauchamp, 1963.** Vue ventrale partielle, montrant la bande marginale crème et grisâtre, et la sole qui légèrement plus pâle le long de la ligne médiane. Échelle : millimètres.

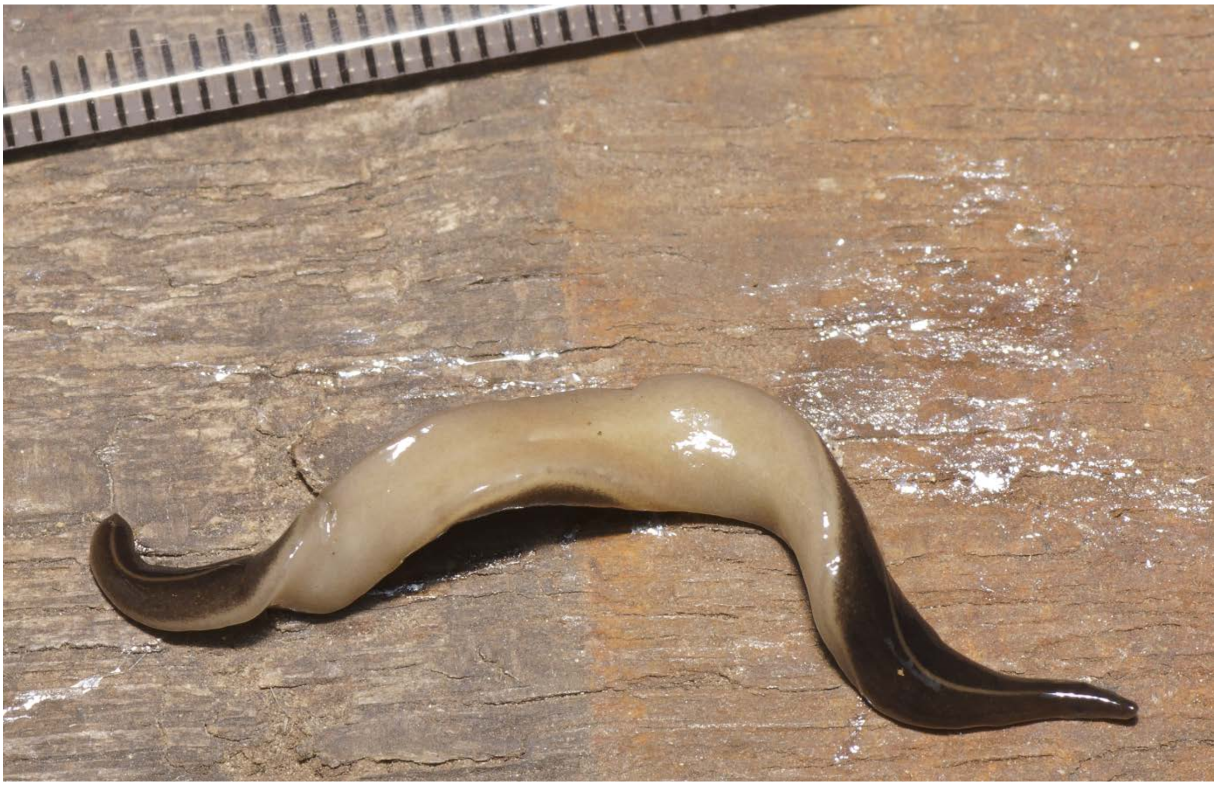

Figure 5. *Platydemus manokwari* de Beauchamp, 1963, prédation expérimentale sur un escargot indigène. Le Plathelminthe est en train de se nourrir de l'escargot : il a été dérangé, ce qui met en évidence le pharynx blanc et cylindrique sortant du ventre, qui fait saillie et ingère des tissus tendres de l'escargot. La proie est l'hélicidé *Eobania vermiculata*, une espèce d'escargot commune de la région méditerranéenne.

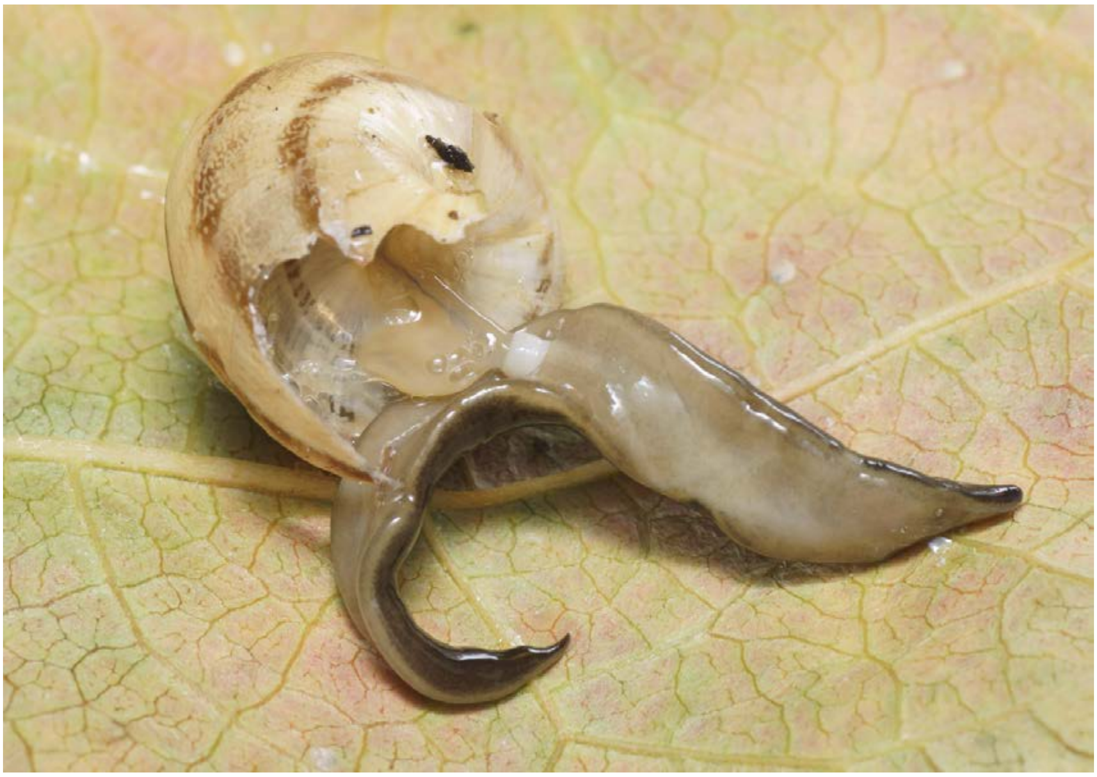

Figure 6. *Platydemus manokwari*, carte mondiale de distribution. Jusqu'à maintenant, *Platydemus manokwari* était confiné dans la région Indo-Pacifique. La mention en France étend cette distribution de manière très significative de la région Indo-Pacifique vers l'Europe.

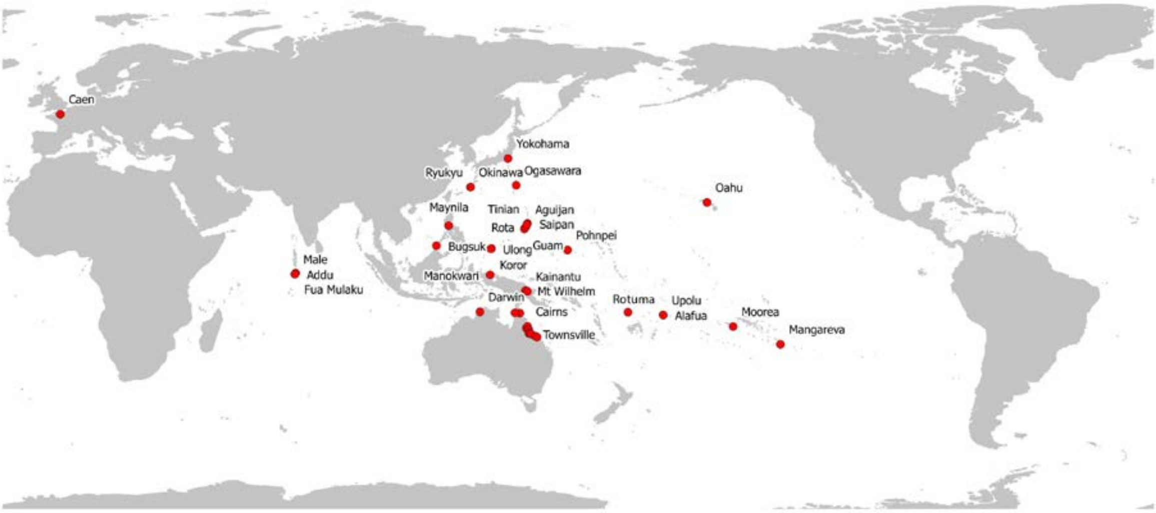

Supplement: Supplemental Information 1 [file peerj-02-297-s001.pdf]
